# Bioimprinting as a Receptor for Detection of Kwakhurin

**DOI:** 10.3390/biom12081064

**Published:** 2022-08-01

**Authors:** Seiichi Sakamoto, Kei Minami, Poomraphie Nuntawong, Gorawit Yusakul, Waraporn Putalun, Hiroyuki Tanaka, Shunsuke Fujii, Satoshi Morimoto

**Affiliations:** 1 Graduate School of Pharmaceutical Sciences, Kyushu University, 3-1-1 Maidashi, Higashi-ku, Fukuoka 812-8582, Japan; south.k.t.4373@gmail.com (K.M.); nutpoomrapee@gmail.com (P.N.); morimoto@phar.kyushu-u.ac.jp (S.M.); 2 School of Pharmacy, Walailak University, Nakhon Si Thammarat 80160, Thailand; gorawit.yu@mail.wu.ac.th; 3 Faculty of Pharmaceutical Sciences, Khon Kaen University, Khon Kaen 40002, Thailand; waraporn@kku.ac.th; 4 Faculty of Pharmaceutical Sciences, Sanyo-Onoda City University, 1-1-1 Daigaku-dori, Yamaguchi 756-0884, Japan; htanaka@rs.socu.ac.jp; 5 Faculty of Health Management, Nagasaki International University, 2825-7 Huis Ten Bosch, Sasebo 859-3298, Japan; fujii@niu.ac.jp

**Keywords:** bioimprinting, enzyme-linked bioimprinted-protein assay (ELBIA), enzyme-linked immunosorbent assay (ELISA), kwakhurin (Kwa), monoclonal antibody (MAb), ovalbumin (OVA)

## Abstract

Bioimprinting was performed against ovalbumin (OVA) to confer its binding cavities for kwakhurin (Kwa), an isoflavonoid, produced solely by *Pueraria candollei* var. *mirifica* (*P. candollei*). The characterization of bioimprinted-OVA (biOVA), evaluated by an enzyme-linked immunosorbent assay (ELISA), revealed that it functioned as a specific receptor for Kwa. Using biOVA, two systems, i.e., an indirect competitive ELISA (icELISA) and the even simpler and more rapid competitive enzyme-linked bioimprinted-protein assay (cELBIA), were developed as novel techniques for the quantitative analysis of Kwa in *P. candollei* and its related products. The two analysis methods were found to have limits of detection (LOD) of 4.0 and 2.5 µg/mL, respectively. The high reliability of the developed icELISA and cELBIA using biOVA was also demonstrated by various validation analyses. Subsequently, bioimprinting was performed using eight other proteins to investigate them as candidate scaffolds for the generation of binding cavities for Kwa. Interestingly, two bioimprinted-IgG monoclonal antibodies (biMAbs) recognized Kwa, but their original binding affinity to hapten was lost. That is, the MAbs obtained a new binding ability to Kwa in exchange for their original binding affinity, raising the possibility that biMAb could be alternatively used as a probe for the quantitative analysis of Kwa as well as biOVA. This is the first report of small molecules recognition by MAbs used as proteins for bioimprinting.

## 1. Introduction

*Pueraria candollei* var. *mirifica* (*P. candollei*), known as “White Kwao Krua” in Thai, is grown in northern and northeastern Thailand. It has a long history as a traditional folk medicine because the extracts from the tuberous roots of *P. candollei* have been shown to possess various pharmacological activities, including anti-osteoporotic [1,2,3], anti-breast cancer [4,5], anti-hypercholesterolemic [6], and anti-oxidant activities [7,8], as well as promoting the improvement of ovariectomy-induced cognitive impairment [8,9]. Among these, the estrogenic activity of *P. candollei* is most prominent because the plant produces potent phytoestrogens. They can be classified into chromenes, such as miroestrol, deoxymiroestrol, and isomiroestrol [10,11,12], and isoflavonoids, such as puerarin, genistin, genistein, daidzin, daidzein, and kwakhurin (Kwa) [13,14].

Recently, health foods and supplements derived from *P. candollei* extracts have attracted much attention among young Japanese women due to attractive catchphrases placed on product packages, including those inferring breast-enhancing and/or body-shaping effects. However, in the case of women with specific physiological symptoms, including menstruation disturbance and metrorrhagia, they have gained the attention of the National Consumer Affairs Center of Japan, and the estrogenic activity of *P. candollei*-derived products is thought to be involved. Therefore, the quality control of *P. candollei*-derived products, based on *P. candollei*-specific phytoestrogens, is required. In our previous study, we focused on Kwa as a marker compound for standardization as it is a phytoestrogen [13] produced solely by *P. candollei*. We also developed various immunoassays for the determination of Kwa in *P. candollei*-derived products [15,16,17,18] as immunoassays enable sensitive and selective analyses of small molecules [19,20]. However, an animal-based approach is necessary for the production of antibodies used in immunoassays.

**Figure 1:**
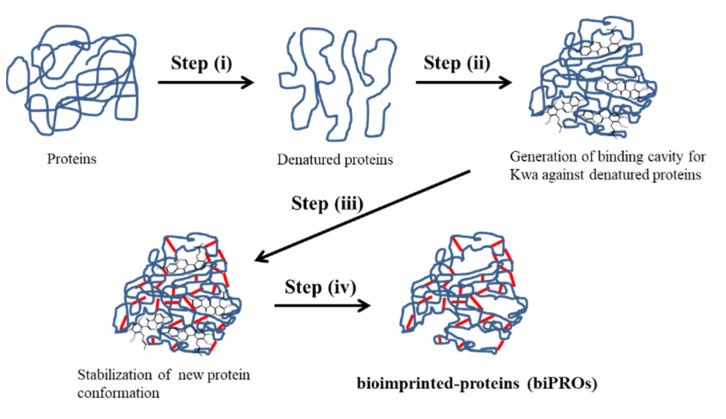
Schematic diagrams of bioimprinting process for Kwa. (i) Proteins are denatured under acidic conditions, and (ii) Kwa is added to the denatured protein for their interaction to generate binding cavity. Subsequently, (iii) denatured proteins are crosslinked with a bifunctional reagent to stabilize the new conformation. Finally, (iv) leftover Kwa template is removed by dialysis to obtain bioimprinted-proteins (biPROs) which recognize Kwa.

Molecular imprinting is a promising approach for the creation of selective recognition sites that are complementary to a template molecule in terms of shape and size. These imprinted sites can act as built-in polymer scaffolds [21] and are initially created via interaction between a monomer and the template molecule. Protein-based bioimprinting was first developed to enhance the catalytic activity of lipase, following the concept of molecular imprinting [22]. Very few studies have been reported regarding the application of bioimprinting to the quantitative analysis of small molecules. Those that have been reported involved the use of ovalbumin (OVA) for the analysis of aflatoxin B1 [23] and bovine serum albumin (BSA) for deoxynivalenol and zearalenone [24,25]. The bioimprinting process for small molecules is relatively simple (Figure 1) and includes the following: (i) denaturation of the initial protein under acidic conditions; (ii) the addition of target template molecules to interact with the denatured protein to form a new molecular configuration; (iii) crosslinking of the protein with a bifunctional reagent, such as glutaraldehyde, to stabilize the new conformation; and (iv) dialysis of the stabilized protein to remove the leftover template.

In the present study, bioimprinting was performed against OVA to confer a binding cavity based on the configuration of Kwa. Using bioimprinted-OVA (biOVA), an indirect competitive enzyme-linked immunosorbent assay (icELISA) and a competitive enzyme-linked bioimprinted-protein assay (cELBIA) were developed as reliable method to detect Kwa in *P. candollei* and its related products. Furthermore, other proteins were subjected to bioimprinting to investigate them as candidate scaffolds for the generation of binding cavities for Kwa.

The preparation of biOVA, its application to icELISA and cELBIA, and findings regarding the potential of bioimprinted-proteins for the determination of Kwa are described herein.

## 2. Materials and Methods

### 2.1. Materials and Reagents

Authentic Kwa (≥96%) was isolated from the tuberous roots of *P. candollei* using the method described in a previous report [15]. OVA (≥98%), BSA (≥97%), human serum albumin (HSA, ≥99%), and *N*,*N′*-carbonyldiimidazole (CDI) were obtained from Sigma-Aldrich (St. Louis, MO, USA). A previously reported anti-glycyrrhizin (GC) monoclonal antibody (MAb 2H2) [26] and anti-harringtonine (HT) MAb (MAb 1D2) [27] were used as candidate proteins for bioimprinting. Anti-OVA antibody (6C8) (ab17293), anti-mouse immunoglobulin G1 (IgG1) goat antibody (horseradish peroxidase, HRP) (ab97240), anti-mouse IgG goat antibody (HRP) (ab6789), and goat F(ab) anti-mouse IgG H&L (HRP) (ab6823) were obtained from Abcam (Cambridge, MA, USA). Glutaraldehyde, used for crosslinking denatured proteins, and an ELISA POD Substrate 3,3,5,5-tetramethylbenzidine (TMB) Kit, used as a substrate solution for ELISA and ELBIA, were obtained from Nacalai Tesque (Kyoto, Japan). All other chemicals were standard commercial products of analytical grade.

Sample preparation for the analysis of Kwa is described in the Supplementary Materials. 

### 2.2. Bioimprinting Procedure

Each protein (1.0 mg) was dissolved in distilled water (1.0 mL) by stirring for 1 min. To denature the protein, the pH of the protein solution was adjusted to ~3 by the addition of HCl (150 µL, 0.1 M), and the solution was stirred for 10 min. To generate a Kwa configuration in the denatured protein, Kwa (100 µL, 200 µg/mL) prepared in 20% (*v*/*v*) methanol was added to the protein solution, and the mixture was stirred for 10 min. After adjusting the pH of the mixture to ~8 by the addition of NaOH (200 µL, 0.1 M), glutaraldehyde (100 µL, 1% (*v*/*v*)) was added to the mixture, which was then stirred at 4 °C for 30 min. The mixtures were statically incubated at 4 °C overnight, and then dialyzed against phosphate-buffered saline (PBS) containing 5% (*v*/*v*) glycerol (GLR) at 4 °C for 48 h to obtain bioimprinted proteins (biPROs). As a negative control, non-biPROs were prepared using 20% (*v*/*v*) methanol (100 µL) instead of a Kwa solution. Both the biPROs and non-biPROs were kept at 4 °C until use.

### 2.3. Preparation of Kwa-HSA and Kwa-HRP Conjugates

**Figure 2:**
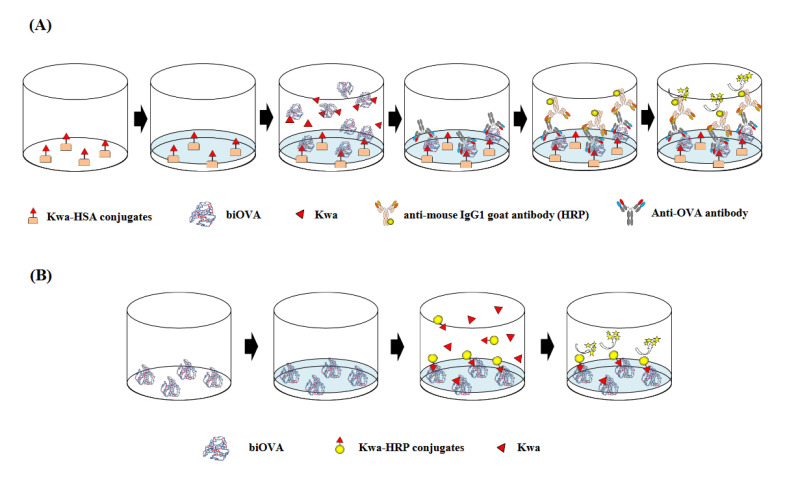
Schematic diagrams of (**A**) the icELISA and (**B**) cELBIA using biOVA. (**A**) The biOVA competitively reacted with the free Kwa or Kwa on the Kwa-HSA conjugates and biOVA binding to Kwa-HSA conjugates were continuously detected by anti-OVA antibody and HRP-labeled anti-mouse IgG1 goat antibody. (**B**) The free Kwa and Kwa-HRP conjugates competitively reacted to the immobilized biOVA. Binding of the Kwa-HRP conjugates to biOVA was detected.

The Kwa-HSA and Kwa-HRP conjugates were used when conducting ELISA (Figure 2A) and ELBIA (Figure 2B), respectively. The zero-length crosslinking reagent CDI was used for conjugation between Kwa and HSA or HRP. The conjugates were prepared using a previously reported method with slight modification [15].

The Kwa (2.5 mg) and CDI (3.4 mg) were dissolved in super dehydrated dimethylformamide (250 µL) and stirred at room temperature for 3 h. Subsequently, the mixture was added to HSA (4.8 mg) solution prepared in a 50 mM carbonate buffer (pH 9.6, 2 mL), and further stirred at room temperature overnight. The solution was then dialyzed against distilled water at 4 °C for 48 h, and lyophilized to yield Kwa-HSA conjugates (3.8 mg). The Kwa-HRP conjugates (3.0 mg) were prepared using the same procedure, except HRP (4.0 mg) was used instead of HSA. Both conjugates were kept at −20 °C until use.

### 2.4. icELISA Using biOVA

An icELISA was used to investigate the binding activity of biOVA against Kwa, which is involved in the ability in the quantitative analysis (Figure 2A). Primarily, Kwa-HSA conjugates (2 µg/mL, 100 µL per well) in a 50 mM carbonate buffer (pH 9.6) were immobilized to the surface of each well of immunoplate (96 Well ELISA Microplate, PS, MICROLON, F-Bottom; Greiner Bio-One, Kremsmünster, Germany). After washing the plate three times with PBS containing 0.05% (*v*/*v*) Tween 20 (PBS-T), a blocking solution (300 µL per well) was added to the well to avoid non-specific adsorption. After the washing step, various concentrations of Kwa, prepared in 5% (*v*/*v*) methanol (50 µL per well) and biOVA (200 µg/mL, 50 µL per well) were added to the wells to evaluate the binding activity against Kwa. The plate was then washed, and anti-OVA antibody (6C8) (100 µL per well), diluted 4,000 times with PBS-T, was added to the wells to detect the biOVA, which was bound to the immobilized Kwa-HSA conjugates. After washing the plate, the anti-mouse IgG1 goat antibody (HRP) (100 µL per well), diluted 20,000 times with PBS-T, was added to each well to detect anti-OVA antibodies (6C8). The plate was then washed. Finally, a TMB substrate solution (100 µL per well) was added, followed by incubation at 37°C for 20 min to develop coloring. The absorbance at 450 nm was subsequently measured using a microplate reader (Multiskan™ FC microplate photometer, Thermo Fisher Scientific, Inc., Waltham, MA, USA). In each step of this assay, incubation was performed at 37 °C for 1 h. 

The cross-reactivities (CRs) of biOVA against various compounds were calculated on the basis of the values obtained from icELISA using the following Equation (1) [28]:

(1) $${\mathrm{CR}}_{s}(\%)=\frac{{\mathrm{IC}}_{50}\mathrm{for}\mathrm{KWa}}{{\mathrm{IC}}_{50}\mathrm{for}\mathrm{compound}\mathrm{under}\mathrm{investigation}}\times 100$$

### 2.5. Indirect ELISA (iELISA) Using biOVA 

To investigate the binding activity of biOVA against immobilized Kwa-HSA conjugates, an indirect ELISA (iELISA) was performed using the same procedure as that of icELISA, except that free Kwa was not used. In iELISA, biOVA (100 µL per well) was applied instead of a mixture of free Kwa (50 µL per well) and biOVA (50 µL per well). Additionally, biOVA prepared without Kwa (non-biOVA) was used as a negative control.

### 2.6. cELBIA Using biOVA

Here, cELBIA was used to investigate the competitive binding activity of the biOVA (Figure 2B). The biOVA (100 µL per well) diluted with a 50 mM carbonate buffer (pH 9.6) was immobilized to the surface of each well of the immunoplate (96 Well ELISA Microplate, PS, MICROLON, F-Bottom; Greiner Bio-One, Kremsmünster, Germany). After washing the plate three times with PBS-T, PBS containing 1% (*w*/*v*) BSA (300 µL per well) was added to each well for blocking. After the washing step, various concentrations of Kwa prepared in 5% (*v*/*v*) methanol (50 µL per well) and Kwa-HRP conjugates (50 µL per well) were added to the wells to compete against the immobilized biOVA. The plate was then washed, and a TMB substrate solution was added, followed by incubation at 37 °C for 20 min for color development. The absorbance at 450 nm was then measured using the MultiskanTM microplate reader. The binding activity of biPROs against Kwa was also evaluated by cELBIA. 

The inhibition rate (IR) of biOVA against Kwa in icELISA and cELBIA was calculated using the following Equation (2):

(2) $${\mathrm{CR}}_{s}(\%)=\frac{A_{0}-A}{A_{0}}\times 100$$

where *A*_0_ is the absorbance without Kwa and *A* is the absorbance with Kwa.

### 2.7. Non-Competitive ELBIA (ncELBIA) Using biOVA

To investigate the binding activity of biOVA against Kwa-HRP conjugates, ncELBIA was performed using the same procedure as for cELBIA, except free Kwa was not used. In ncELBIA, Kwa-HRP conjugates (100 µL per well) were applied to each well instead of a mixture of free Kwa (50 µL per well) and Kwa-HRP conjugates (50 µL per well). Additionally, non-biOVA was used as a negative control. 

## 3. Results

### 3.1. Development of the icELISA and cELBIA Using biOVA

#### 3.1.1. Optimization of Various Parameters for icELISA

The Kwa-HSA conjugates, used as immobilized proteins for ELISA, were prepared using a CDI-mediated method. The number of Kwa binding to HSA was evaluated by matrix-assisted laser desorption/ionization time-of-flight mass spectrometry (MALDI-TOF-MS, Bruker Autoflex III). The results showed that at least three molecules were bound to HSA molecules; the molecular weights of the Kwa-HSA conjugates, HSA, and Kwa were found to be 67,680, 66,518, and 368.39, respectively (Supplementary Figure S1).

**Figure 3:**
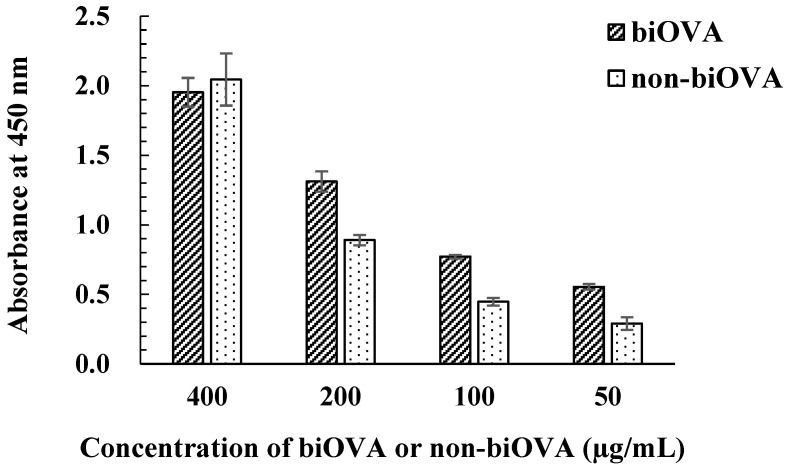
Optimization of biOVA concentration. biOVA and non-biOVA were diluted with PBS-T (50, 100, 200, and 400 µg/mL) and applied to iELISA.

The concentration of biOVA was first optimized using the iELISA with biOVA and non-biOVA (Figure 3). Both biOVA and non-biOVA were diluted with PBS-T (50, 100, 200, and 400 µg/mL) and applied to iELISA. As a result, obvious differences in absorbance appeared at 50, 100, and 200 µg/mL. When biOVA was used at 200 µg/mL, the absorbance value at 450 nm was above 1.0. Since an absorbance of ~1.0 in iELISA provides reliable data for analysis using icELISA [29], this biOVA concentration was selected as the optimal concentration for the ELISA.

Immunoplates were then optimized to decrease the background derived from the non-specific adsorption of biOVA and non-biOVA (Supplementary Figure S2). When seven plates were evaluated, 96 Well ELISA Microplate PS, MICROLON, F-Bottom (greiner bio-one, Kremsmünster, Germany) and SpectraPlate-96 Medium protein binding affinity (PerkinElmer, Inc., Waltham, MA, USA) exhibited obvious differences in absorbance between biOVA and non-biOVA. However, the former plate was selected for further ELISA testing due to its higher absorbance value.

Interestingly, it was revealed that the blocking step was unnecessary for the icELISA using biOVA; this was because the lowest non-specific adsorption and highest IR against free Kwa were obtained using a non-blocked plate (Supplementary Figure S3).

These optimizations revealed that the icELISA using biOVA could be completed in ~4.5 h with five steps.

#### 3.1.2. Optimization of Various Parameters for cELBIA

The concentrations of biOVA and Kwa-HRP conjugates were optimized using ncELBIA with various biOVA concentrations (100, 200, and 300 µg/mL) and Kwa-HRP conjugates (0.01–100 µg/mL) (Supplementary Figure S4), and the combination of the lowest concentrations at which the absorbance was ~1.0 was selected for cELBIA. As a result, the optimal concentrations of biOVA and Kwa-HRP conjugate were found to be 300 µg/mL and 50 µg/mL, respectively.

In ELBIA, the obvious non-specific adsorption of biOVA and non-biOVA was observed without a blocking step (Supplementary Figure S5). Therefore, blocking was performed using PBS containing 1% (*w*/*v*) BSA.

These optimizations revealed that the cELBIA using biOVA could be completed in ~3.5 h with four steps.

#### 3.1.3. Characterization of biOVA

**Figure 4:**
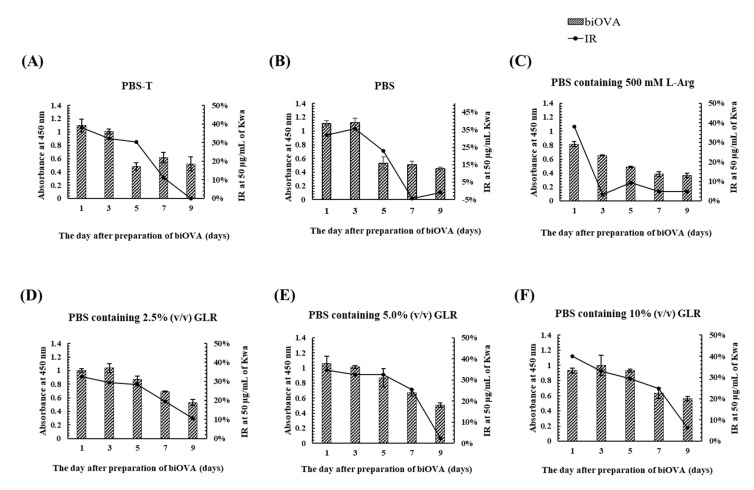
The effect of various dialysis buffers on the stability of biOVA evaluated by iELISA and icELISA. (**A**) PBS-T, (**B**) PBS, (**C**) PBS containing 500 mM Arg, (**D**) PBS containing 2.5% (*v*/*v*) GLR, (**E**) PBS containing 5.0% (*v*/*v*) GLR, and (**F**) PBS containing 10% (*v*/*v*) GLR were used as dialysis buffer. Free Kwa (50 μg/mL) was used as a competitive compound for icELISA.

To investigate the effects of a dialysis buffer on the stability of biOVA during its preparation, various PBS-based dialysis buffers (PBS-T, PBS containing 500 mM L-arginine (Arg), and PBS containing 2.5–10% (*v*/*v*) GLR) were used at 4 °C during the final step of biOVA preparation. Furthermore, time-dependent absorbance values and IR were evaluated by iELISA and icELISA, respectively, performed at 1, 3, 5, 7, and 9 days after preparation of the biOVA (Figure 4). The absorbances for PBS-T, PBS, and PBS containing Arg decreased at 5 days, whereas both the absorbance and IR for PBS containing 2.5–10% (*v*/*v*) GLR decreased at 7 days. When the changes in IR from 5 to 7 days were compared among three GLR concentrations, the IR was found to decrease by ~10% for PBS containing 2.5% (*v*/*v*) GLR, whereas it decreased by ~5% for the others. Because a similar pattern was observed for PBS containing 5% and 10% (*v*/*v*) GLR, PBS containing 5% (*v*/*v*) GLR was selected as a dialysis buffer. These results also indicated that the biOVA was stable for 5 days at 4 °C when PBS containing 2.5–10% (*v*/*v*) GLR was used as a dialysis buffer.

The specificity of biOVA was evaluated by icELISA with various 12 compounds, including structurally related isoflavones (Table 1). Relatively high CRs were observed against the isoflavonoids daidzein and genistein, at 23.4% and 25.5%, respectively, whereas negligible CRs were observed against their glycosides daidzin and genistin at 9.4% and 5.2%, respectively. No CRs were observed against the remaining eight compounds.

#### 3.1.4. Determination of Kwa by the icELISA and cELBIA Using biOVA

**Figure 5:**
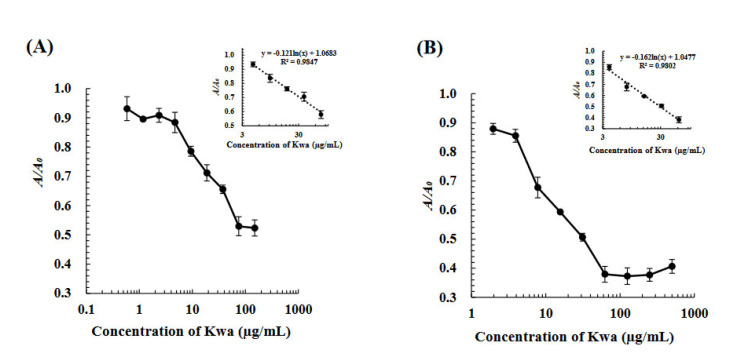
Calibration curves of (**A**) the icELISA and (**B**) cELBIA using biOVA. In icELISA, Kwa-HSA conjugates and biOVA were used at 2 and 200 μg/mL, respectively. In cELBIA, Kwa-HRP conjugates and biOVA were used at 50 and 300 μg/mL, respectively.

Under optimized conditions, the icELISA and cELBIA using biOVA were developed for quantitative analysis. Standard calibration curves for the determination of Kwa were obtained using various concentrations of Kwa, as shown in Figure 5. For icELISA, Kwa could be detected in the range of 4.7–75.0 µg/mL with a limit of detection (LOD) of 4.0 µg/mL for icELISA. For cELBIA, Kwa could be detected in the range of 3.9–62.5 µg/mL with an LOD of 2.5 µg/mL.

The reliability of the icELISA using biOVA was also supported by measurement of their intra- and inter-assay precision (Supplementary Table S1).

Subsequently, the amounts of Kwa in *P. candollei* and its related products were determined by icELISA and cELBIA developed using biOVA and then compared with those determined by the icELISA using MAb 11F (Table 2) [15]. The results revealed that Kwa was not detected in Figures S1 or S5 because the amount was less than minimum limit of determination. 

### 3.2. Investigation of Candidate Proteins for the Bioimprinting of Kwa

**Figure 6:**
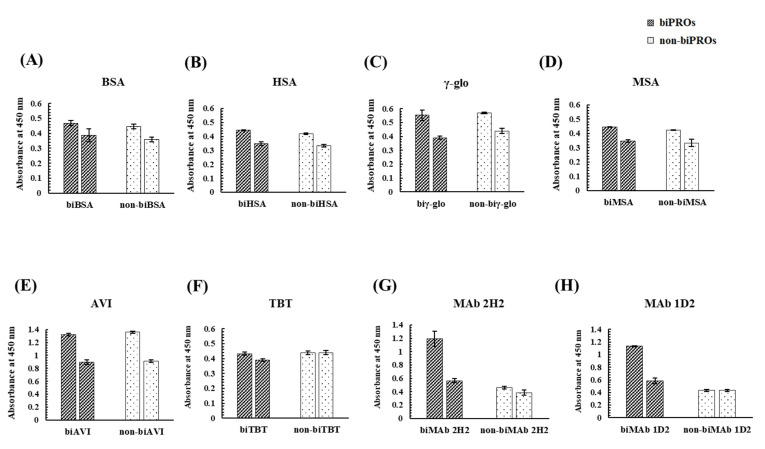
Evaluation by ELBIA of the binding activity of the biPROs and non-biPROs of eight proteins to Kwa: (**A**) BSA, (**B**) HSA, (**C**) γ-glo, (**D**) MSA, (**E**) AVI, (**F**) TBT, (**G**) MAb 2H2, and (**H**) MAb 1D2.

To investigate whether proteins other than OVA could obtain a binding ability to Kwa, bioimprinting was performed against eight candidate proteins, i.e., BSA, HSA, γ-globulin from human serum (γ-glo), mouse serum albumin (MSA), avidin from egg white (AVI), thyroglobulin from bovine thyroid (TBT), MAb 2H2 [26], and MAb 1D2 [27]. The binding activities of biPROs/non-biPROs to Kwa-HRP conjugates were evaluated by ncELBIA, while those to free Kwa were evaluated by cELBIA (Figure 6). In the bar graphs for each biPRO and non-biPRO, the left graph represents the absorbance of the ncELBIA, while the right graph indicates that of cELBIA using free Kwa at 25 μg/mL.

As a result, almost identical absorbances were obtained from biPROs and non-biPROs when BSA, HSA, γ-glo, MSA, and AVI were subjected to bioimprinting. In addition, similar inhibitory activities of the five proteins (BSA, HSA, γ-glo, MSA, and AVI) against Kwa were observed in the biPROs and non-biPROs, indicating that the non-specific adsorption between Kwa and the immobilized biPROs and non-biPROs were occurred. As for TBT, no differences in absorbance between biTBT and non-biTBT and those between ncELBIA (left graph) and cELBIA (right graph) were observed. Interestingly, the biPROs of MAb 2H2 and MAb 1D2, which functioned against GC and HT, respectively, exhibited high binding activity against free Kwa with IRs of 53% and 49%, respectively (Figure 6G,H). 

**Figure 7:**
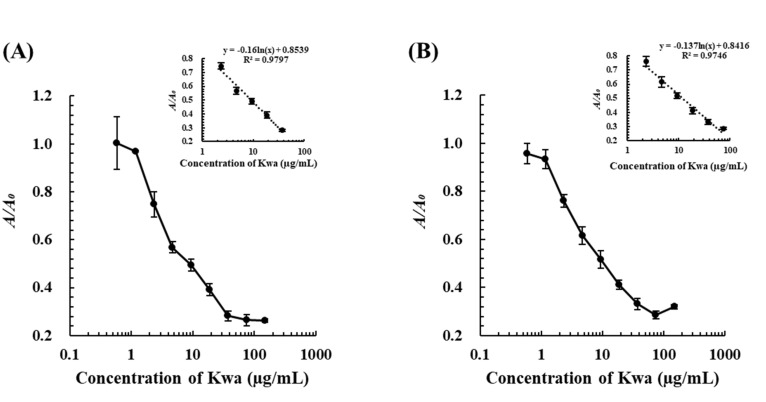
Calibration curves of cELBIA using (**A**) biMAb 2H2 and (**B**) biMAb 1D2. The Kwa-HRP conjugates and biMAbs (biMAb 2H2 and biMAb 1D2) were used at 50 and 225 μg/mL, respectively.

Standard calibration curves by cELBIA using biMAb 2H2 and biMAb 1D2 were obtained under optimized conditions (Supplementary Figure S6) for Kwa determination. In this test, the detectable range of Kwa by the cELBIA using biMAb 2H2 was 2.3–37.5 µg/mL and had an LOD of 0.8 µg/mL, whereas that using biMAb 1D2 was 2.3–75.0 µg/mL with an LOD of 0.7 µg/mL (Figure 7). These results revealed that the sensitivity of the cELBIA using biMAb 2H2 and biMAb 1D2 was 3–4 times greater than that using biOVA (LOD: 2.5 µg/mL). When the selectivity was evaluated by cELBIA, biMAb 2H2 was found to be more specific to free Kwa compared with biMAb 1D2 (Table 3) and biOVA (Table 1), indicating that an even more specific and sensitive cELBIA could be developed using biMAb 2H2, rather than using biOVA.

**Figure 8:**
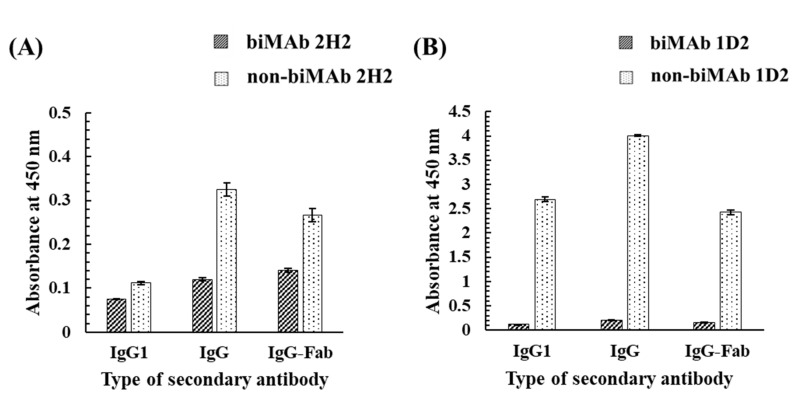
The effect of bioimprinting on the binding ability of (**A**) MAb 2H2 and (**B**) MAb 1D2 to GC and HT, evaluated by iELISA. The GC-HSA and HT-HSA conjugates (2 μg/mL) were immobilized to the plate, and biMAb 2H2 or biMAb 1D2 (300 μg/mL) was applied after blocking; IgG1, IgG, and IgG-Fab represent anti-mouse IgG1 goat antibody (HRP), anti-mouse IgG goat antibody (HRP), and goat F(ab) anti-mouse IgG H&L (HRP), respectively, were used as a secondary antibody.

As noted, MAb 2H2 and MAb 1D2 are IgG1 MAbs that are specific to GC and HT, respectively [26,27]. To investigate the effects of bioimprinting on their activity as antibodies, biMAb 2H2 and biMAb 1D2 were applied to an indirect ELISA, in which GC-HSA and HT-HSA conjugates were used as immobilized antigens (Figure 8). As for secondary antibodies, anti-mouse IgG1 goat antibody (HRP), anti-mouse IgG goat antibody (HRP), and goat F(ab) anti-mouse IgG H&L (HRP) were used against the biMAbs (biMAb 2H2 and biMAb 1D2) and non-biMAbs (non-biMAb 2H2 and non-biMAb 1D2) which were used as primary antibodies. Interestingly, the results for the biMAbs revealed no reactivity, whereas reactivity was found among the non-biMAbs, meaning that bioimprinting caused MAb 2H2 and MAb 1D2 to lose their original ability to bind to GC and HT, respectively, in exchange for the new binding ability to Kwa.

## 4. Discussion

### 4.1. Development of the icELISA and cELBIA Using biOVA for Quantitative Analysis of Kwa

Bioimprinting is one of the promising techniques to confer the binding cavity to the biomolecules, such as proteins. OVA was subjected to bioimprinting under the presence of Kwa to generate biOVA, which was subsequently applied to the icELISA and cELBIA for the quantitative analysis of Kwa in *P. candollei* and its related products.

Characterization of biOVA revealed that biOVA was stable for 5 days at 4 °C under the optimized dialysis buffer which was PBS containing 5% (*v*/*v*) GLR (Figure 4E). Specificity of biOVA is one of the most important factors to develop specific assay. When CRs of biOVA was evaluated by icELISA, biOVA was found to slightly recognized daidzein and genistein, whereas negligible CRs were confirmed for other compounds including their glycosides (Table 1). These results indicated that biOVA was relatively specific to isoflavones, suggesting that the OVA generated a scaffold, based on the Kwa configuration, although its refinement was inferior to that of MAb against Kwa (MAb 11F) [15]. These results also indicated that biOVA could be applied to the quantitative analysis of Kwa.

The icELISA (Figure 2A) was primarily developed. Optimization of various parameters for icELISA revealed that the optimal concentration of biOVA for icELISA was found to be 200 µg/mL. When plate was optimized, non-specific adsorption of non-biOVA was observed in the five plates used (Supplementary Figure S2B–F), indicating that plate optimization is necessary for developing icELISA. Interestingly, the lowest non-specific adsorption of biOVA/non-biOVA and the highest IR against Kwa were obtained, which suggested that the blocking step was not mandatory step. This result was in agreement with our previous reports regarding development of magnetic particles-based enzyme immunoassay [17,30]. Furthermore, Ahirwar et al. reported unnecessity of BSA blocking step in common ELISA protocol [31]. Since rapid analysis may be developed, the necessity of the blocking step must be evaluated when developing a new enzyme immunoassay.

The cELBIA was then developed. Optimization of various parameters for cELBIA revealed that the optimal concentrations of biOVA and Kwa-HRP conjugate were found to be 300 µg/mL and 50µg/mL, respectively, and the blocking step was necessary for developing cELBIA using biOVA.

It was revealed LOD for Kwa detection of icELISA and cELBIA exhibited 4.0 and 2.5 µg/mL, respectively. The sensitivities of the developed icELISA and cELBIA using biOVA were found to be low compared with those of the icELISA using MAb 11F, which showed an LOD of 1.1 ng/mL [15]. However, different application of biPROs could enable the development of more sensitive quantitative analysis. Gutierrez et al. reported current pulse capacitive measurement using biOVA that recognized aflatoxin B1 at a detectable range of 1 ng/mL to 1 µg/mL [23]. Furthermore, Beloglazova et al. reported the application of bioimprinted-BSA to a multiplex assay for the detection of zearalenone and deoxynivalenol in agricultural products with detectable ranges of 10–290 ng/mL and 55–420 ng/mL, respectively [24]. The sensitivity can also be improved by applying biPROs to other detection systems.

When the Kwa amounts determined by the icELISA and cELBIA using biOVA were compared with those determined by the icELISA using MAb 11F, most Kwa amounts determined by the biOVA-based methods exhibited higher values than those determined by the icELISA using MAb 11F. These results accounted for the wide CRs of biOVA, which also recognized *P. candollei*-derived isoflavonoids, such as daidzein and genistein. Nonetheless, the newly developed assays using biOVA were found to be reliable because the measured Kwa amounts were close to those determined by the icELISA using MAb 11F.

### 4.2. Investigation of Candidate Proteins for the Bioimprinting of Kwa

In this study, biOVA exhibited superior characteristics to Kwa as compared with other biPROs except biMAbs. Under the acidic condition, predominant interactions between protonated Kwa and denatured proteins are hydrophobic interactions rather than electrostatic interactions [25]. Therefore, these results are accounted for by the hydrophobicity of each protein. Physicochemical properties of OVA, BSA, HSA, γ-glo, MSA, AVI, and TBT computed by Expasy’s Protparam server (http://web.expasy.org/protparam/) (Accessed on 27 July 2022) revealed that OVA shows the highest aliphatic index [32] and grand average of hydropathicity (GRAVY) index [33] (Supplementary Table S2), which are involved in hydrophobicity and hydrophilicity. Since these results suggested that OVA is more hydrophobic than others, OVA interacted predominantly with Kwa to form binding cavities, and resultant biOVA exhibited binding activity against free Kwa.

The MAb 2H2 and MAb 1D2 showed potential to be used as probes for bioimprinting. So far, the proteins used for bioimprinting have been limited to BSA and OVA [23, 24, 25]. Since physicochemical properties of the two were not obviously different from other proteins (Supplementary Table S2), other factors may cause changes in configuration suitable for binding of Kwa. MAb 2H2 and MAb 1D2 are IgG molecules which are generally produced by the immune system. Therefore, they may have a structure to easily access to molecule even after denaturation and crosslinking. Our findings indicated that the MAbs could be used as probes for bioimprinting, which could be applied to the quantitative analysis of Kwa as well as OVA. It is worth noting that this is the first report of small molecules recognition by MAbs used as proteins for bioimprinting.

## 5. Conclusions

In this study, bioimprinting was performed against OVA to confer its binding cavities for Kwa to develop quantitative analysis of Kwa. Using biOVA, icELISA, and cELBIA, we successfully developed a reliable method. When bioimprinting was performed against various proteins, MAbs (MAb 2H2 and MAb 1D2), as well as OVA, were found to recognize Kwa. Moreover, the cELBIA using biMAb 2H2 exhibited greater specificity and sensitivity than when using biOVA. This study posits the novel proposal that MAb can be used as a probe for bioimprinting for small molecules. To date, very few studies have been reported on biPROs that recognize small molecules [23,24,25]. Additionally, in-depth studies are needed as bioimprinted probes can easily be prepared and used in a range of contexts, such as diagnoses, medicine, and general research.

## Figures and Tables

**Table 1:** The CRs of biOVA against major isoflavones, evaluated by icELISA.

		CRs (%)	
**Class**	**Compound**	**biOVA**	**MAb 11F ^a^**
Isoflavonoids	Kwa	100	100
	Daidzein	23.4	<0.005
	Genistein	25.5	<0.005
Isoflavonoid glycosides	Daidzin	9.4	<0.005
	Genistin	5.2	<0.005
	Puerarin	<0.1	<0.005

^a^ The CRs of MAb 11F were evaluated by icELISA [15].

**Table 2:** The determination of Kwa by the icELISA and cELBIA using biOVA in *P. Candollei*-derived samples and products.

Sample Name	icELISA Using biOVA		cELBIA Using biOVA		icELISA Using MAb 11F	
	Kwa Amount (% wt./dry wt.)	CV (%)	Kwa Amount (% wt./dry wt.)	CV (%)	Kwa Amount (% wt./dry wt.)	CV (%)
*P. candollei* root without bark	4.23 × 10^−4^	11.8	4.49 × 10^−4^	9.0	1.94 × 10^−4^	7.0
*P. candollei * root bark 1	1.56 × 10^−2^	12.1	1.43 × 10^−2^	7.2	1.09 × 10^−2^	8.2
*P. candollei * root bark 2	1.22 × 10^−3^	6.2	1.11 × 10^−3^	9.3	1.29 × 10^−3^	0.4
Supplement 1	9.19 × 10^−5^	4.3	ND	ND	9.94 × 10^−6^	10.3
Supplement 2	6.89 × 10^−3^	11.7	9.65 × 10^−3^	4.9	6.23 × 10^−3^	4.5
Supplement 3	2.04 × 10^−3^	8.1	2.14 × 10^−3^	9.3	1.27 × 10^−3^	5.0
Supplement 4	3.36 × 10^−4^	8.7	4.12 × 10^−4^	6.5	2.38 × 10^−4^	9.0
Supplement 5	ND	ND	ND	ND	5.73 × 10^−6^	9.4

CV and ND represent the coefficient of variation and “not detected”, respectively. All values are mean ± standard deviation (SD) from triplicate samples.

**Table 3:** The CRs of biMAb 2H2 and biMAb 1D2 against major isoflavones, evaluated by cELBIA.

		CRs (%)	
Class	Compound	biMAb 2H2	biMAb 1D2
Isoflavonoids	Kwa	100	100
	Daidzein	<0.1	0.2
	Genistein	<0.1	7.1
Isoflavonoid glycosides	Daidzin	<0.1	5.4
	Genistin	0.1	19.6
	Puerarin	7.0	57.3

## Data Availability

All the data supporting the findings of this study are included in this article.

## Supplementary Materials

The following supporting information can be downloaded at: https://www.mdpi.com/article/10.3390/biom12081064/s1, Figure S1: Evaluation of Kwa number on the Kwa-HSA conjugates by MALDI-TOF-MS analysis; Figure S2: Optimization of the plate for the icELISA using biOVA and non-biOVA; FigureS3: Optimization of the blocking solution; Figure S4: The optimization of biOVA and Kwa-HRP conjugates concentrations; Figure S5: Evaluation of the necessity of the blocking step in ELBIA using (A) a non-blocked plate and (B) a blocked plate with PBS containing 1% (*v*/*w*) BSA; Figure S6: Optimization of concentration of biMAbs (A) biMAb 2H2 and (B) biMAb 1D2 and Kwa-HRP conjugates. Table S1: Intra- and inter-assay precision tests of (A) icELISA and (B) cELBIA using biOVA. Table S2: Physicochemical properties of OVA, BSA, HSA, γ-glo, MSA, AVI, TBT, MAb 2H2, and MAb 1D2.
