# Supplementary material for: Bioimprinting as a Receptor for Detection of Kwakhurin
Source: Biomolecules. 2022 Aug 1;12(8):1064. doi: 10.3390/biom12081064 (PMC9405580; doi:10.3390/biom12081064)
Supplement: Supplementary file 1 [file biomolecules-12-01064-s001.zip › biomolecules-1843988-supplementary.pdf]

Sample preparation for the analysis of Kwa

Three *P. candollei*-derived plant samples (*P. candollei* root without bark, *P. candollei* root bark 1, and *P. candollei* root bark 2) and Five *P. candollei*-derived products (supplements 1–5) were used. Sample preparation for quantitative analysis of Kwa in *P. candollei*-derived samples and products was performed by our previous report with slight modification [15]. 250 mg of *P. candollei*-derived samples and products were sonicated with methanol (1 mL) for 30 min. After centrifugation at 10,000 rpm for 10 min, supernatant was transferred into small test tubes. This extraction step was repeated more four times, and resulting methanol extracts (5 mL) was removed under nitrogen gas. Finally, methanol (50 µL) was added to the residues and kept at –20°C until use.

To evaluate number of Kwa binding to HSA was evaluated by MALDI-TOF-MS analysis (Bruker Autoflex III). The Kwa-HSA conjugates and HSA (1–10 pmol) were mixed with the matrix solution, which consists of saturated sinapinic acid in 0.15% (v/v) trifluoroacetic acid aqueous solution and acetonitrile in a ratio of 2 to 1. The mixture (2 µL) was then spotted onto an MTP 384 ground steel target plate (Bruker Daltonics, Bremen, Germany). The spectra were obtained in positive high-mass mode and analyzed using flexControl software (Bruker Daltonics, Bremen, Germany).

(A)

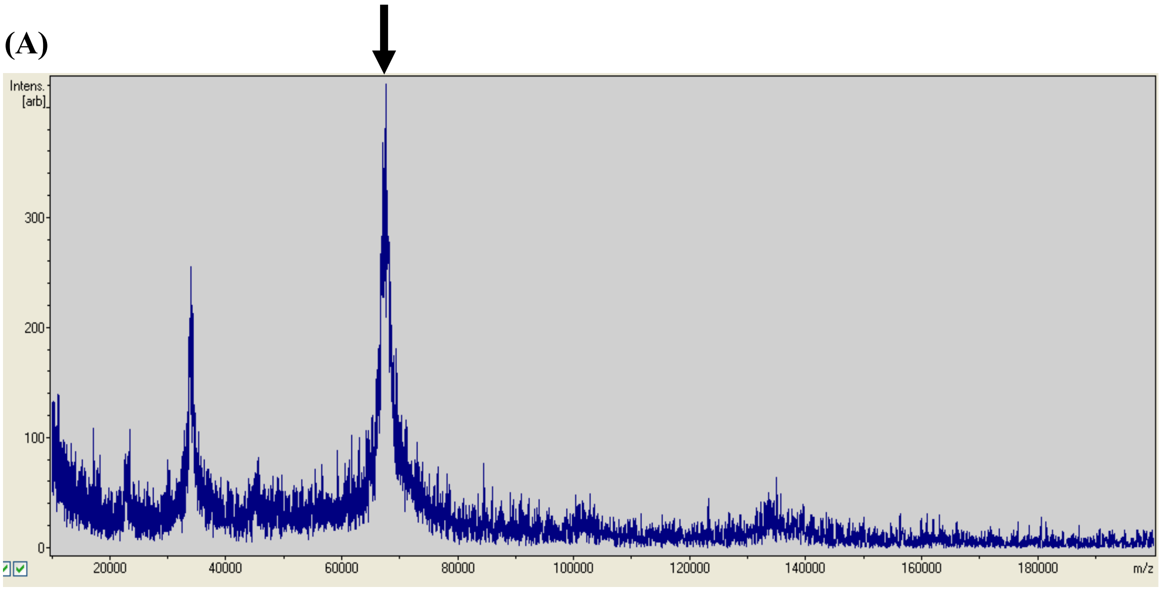

(B)

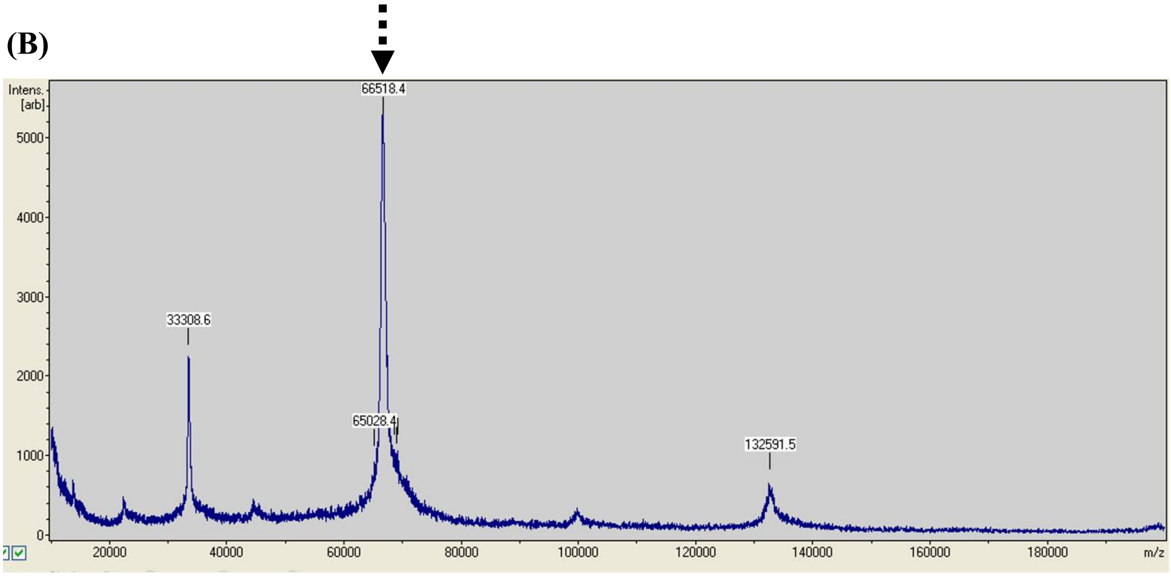

Figure S1. Evaluation of Kwa number on the Kwa-HSA conjugates by MALDI-TOF-MS analysis. MALDI-TOF-MS data for (A) Kwa-HSA conjugates and (B) HSA. Solid and dotted lines represent the peaks of Kwa-HSA conjugates (67,680) and HSA (66,518), respectively.

The immunoplates were optimized to decrease the background derived from the non-specific adsorption of biOVA and non-biOVA (Figure S2). In this test, the Kwa-HSA conjugates-immobilized and non-immobilized areas were prepared in the same plate, and iELISA was performed for seven types of immunoplates using biOVA and non-biOVA (Figure S2).

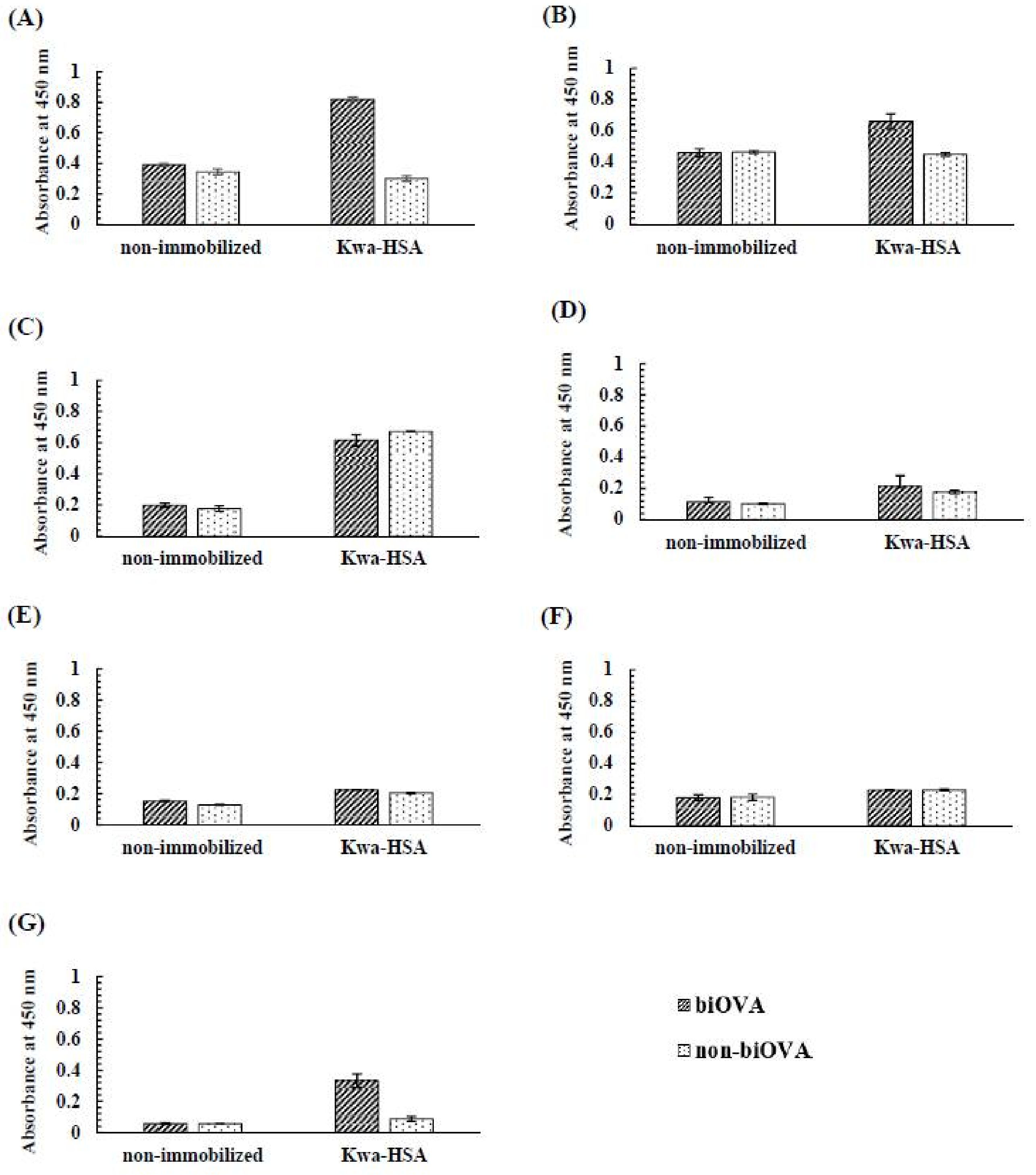

Figure S2. Optimization of the plate for the icELISA using biOVA and non-biOVA. Seven plates were used to evaluate the non-specific adsorption of biOVA and non-biOVA against the palte as follows: (A) 96 Well ELISA Microplate, PS, MICROLON®, F-Bottom; (greiner bio-one, Kremsmünster, Germany); (B) H-type ELISA plate (Sumitomo Bakelite Co., Ltd.,

Tokyo, Japan); (C) ELISA PLATE 96 Well Flat Bottom (IWAKI AGC Techno Glass Co., Ltd., Shizuoka, Japan); (D) Test plate for ELISA 96Well (BM Equipment Co., Ltd., Tokyo, Japan); (E) SpectraPlate-96 High protein binding affinity (PerkinElmer, Inc., Waltham, MA, USA); (F) Corning® 96-well EIA/RIA Clear Round Bottom Polystyrene High Bind Microplate without lid (Corning International Co., Ltd., Osaka, Japan), and (G) SpectraPlate-96 Medium protein binding affinity (PerkinElmer, Inc., Waltham, MA, USA).

To evaluate the non-specific adsorption of biOVA and non-biOVA against the blocking solution, various blocking solutions, 2.5%–10% (w/v) skim milk in PBS, 0.75%–3% (w/v) BSA in PBS, and 1.5%–6% (w/v) gelatin (Gel) in PBS were applied to iELISA without immobilization of Kwa–HSA conjugates (Figure S3A). In this test, a non-blocked plate was also prepared for comparison. All of the skim milk, as well as the 3% and 6% (w/v) Gel, exhibited higher non-specific adsorption compared with other solutions, and the lowest non-specific adsorption was observed when the non-blocked plate was used.

Subsequently, the blocking solution was further optimized using 0.75%–3% (w/v) BSA in PBS, 1.5% (w/v) Gel, and a non-blocked plate for both iELISA and icELISA (Figure S3B). iELISA revealed obvious differences in absorbance between biOVA and non-biOVA. In addition, the highest IR was obtained against Kwa in icELISA when the non-blocked plate was used.

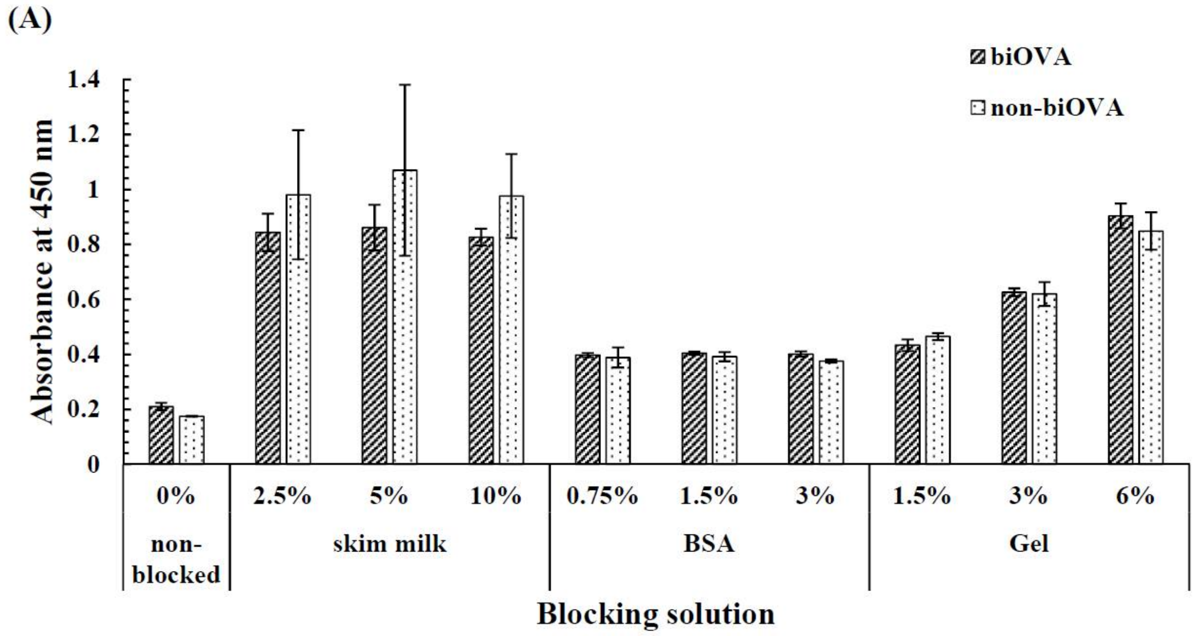

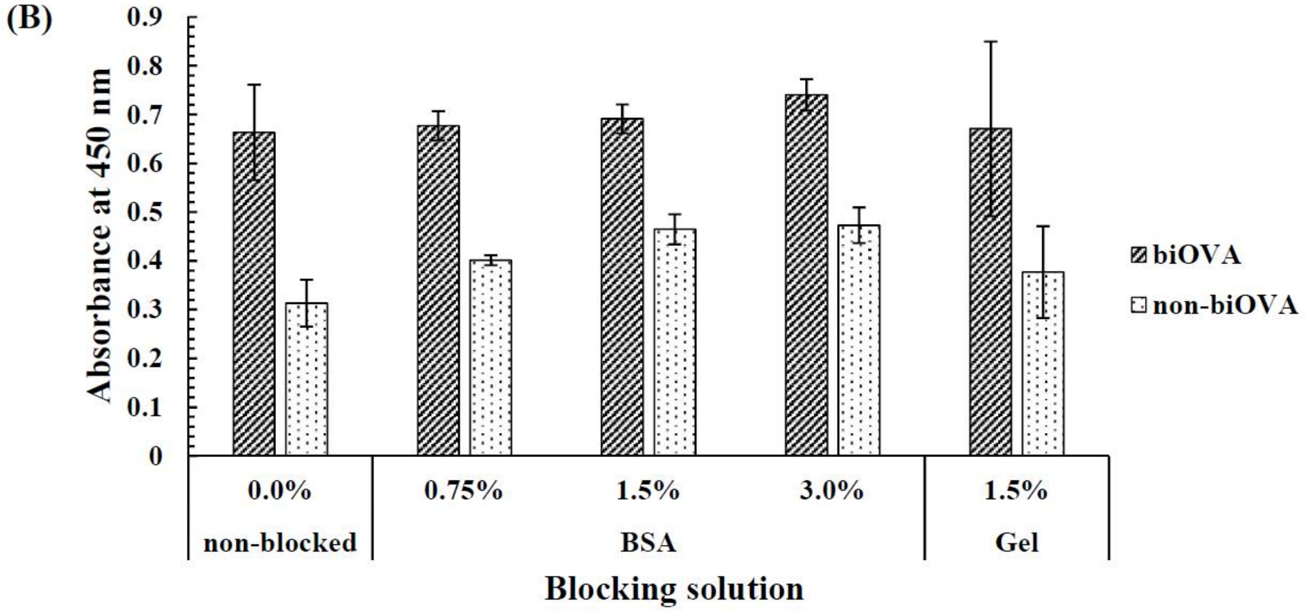

IR	41%	13%	11%	14%	17%

Figure S3. Optimization of the blocking solution. (A) The iELISA with various blocking solutions, including 2.5%–10% (w/v) skim milk in PBS, 0.75%–3% (w/v) BSA in PBS, and 1.5%–6% (w/v) gelatin (Gel) in PBS, without immobilization of Kwa-HSA conjugates. (B) The iELISA and icELISA using 0.75%–3% (w/v) BSA in PBS and 1.5% (w/v) Gel.

The concentrations of biOVA and Kwa-HRP conjugates were optimized by ncELBIA using various concentrations of biOVA and Kwa-HRP conjugates (Figure S4).

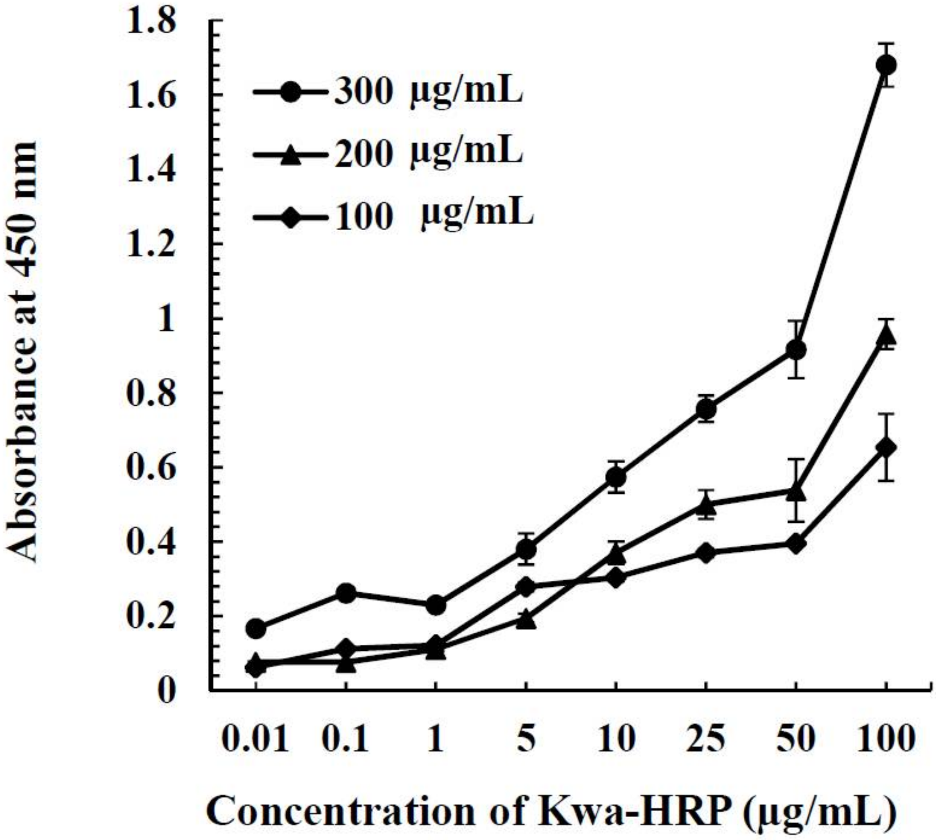

Figure S4. The optimization of biOVA and Kwa-HRP conjugates concentrations. Various concentrations of biOVA (100, 200, and 300 μg/mL) and Kwa-HRP conjugates (0.01–100 μg/mL) were applied to ncELBIA.

To evaluate the necessity of the blocking step in ELBIA, ncELBIA was performed using a non-blocked plate and a blocked plate with PBS containing 1% (v/w) BSA (Figure S5). In both the non-blocked and blocked plates, the absorbances using biOVA and non-biOVA increased as the concentration of Kwa-HRP conjugates increased. However, the absorbances in the blocked plate using biOVA was obviously higher than those using non-biOVA, which was used as a negative control, indicating that a biOVA-specific assay against Kwa could be performed using a blocked plate.

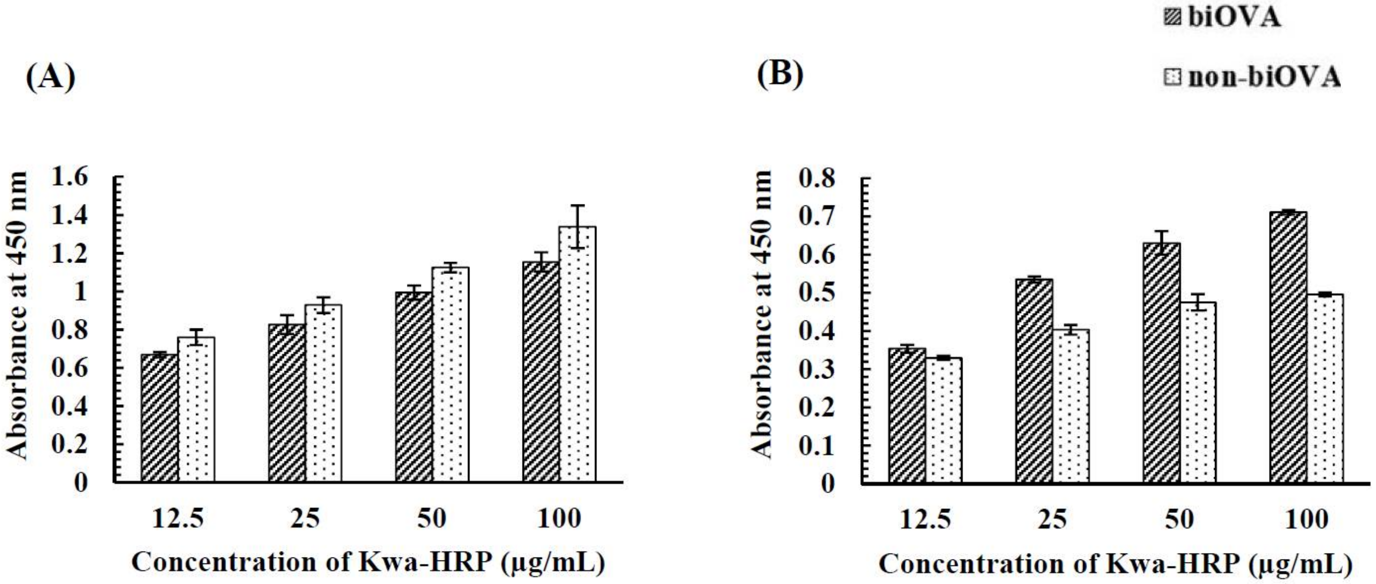

Figure S5. Evaluation of the necessity of the blocking step in ELBIA using (A) a non-blocked plate and (B) a blocked plate with PBS containing 1% (v/w) BSA.

Intra-, and inter-assay precision tests were performed to evaluate the reliability of icELISA and cELBIA. These precisions were evaluated on the basis of the maximum coefficient of variation (CV) obtained from well-to-well and plate-to-plate tests, respectively. The maximum CVs of intra- and inter-assay precision in icELISA were 7.4% and 7.5%, respectively, and those in cELBIA were 8.7% and 7.0%, respectively, indicating that both assays using biOVA are sufficiently reliable for quantitative analysis.

Table S1. Intra- and inter-assay precision tests of (A) icELISA and (B) cELBIA using biOVA.

(A)

Kwa concentration (μg/mL)	CV (%)[a]	
	Intra-assay (n=6)	Inter-assay (n=3)
**75.0**	6.5	2.6
**37.5**	3.1	7.5
**18.8**	3.0	3.2
**9.4**	1.5	4.9
**4.7**	7.4	3.3

(B)

Kwa concentration (μg/mL)	CV (%)[a]	
	Intra-assay (n=6)	Inter-assay (n=3)
**62.5**	8.7	5.6
**31.2**	4.6	6.7
**15.6**	4.6	6.0
**7.8**	7.4	7.0
**3.9**	6.4	3.8

[a] All values represent CV calculated using following formula:

$$\mathbf{CV}\ (\%) = \frac{\text{standard deviation (SD)}}{\text{mean}} \times 100$$

The concentrations of biOVA and Kwa-HRP conjugates were optimized by the ncELBIA using various concentrations of biMAbs (180, 225, 300 and 450 μg/mL) and Kwa-HRP conjugates (6.25–100 μg/mL) (Figure S6). The combination of the lowest concentrations at which the absorbance was ~1.0 was selected for cELBIA. As a result, optimal concentrations of biMAbs (biMAb 2H2 and biMAb 1D2) and Kwa-HRP conjugate were found to be 225 and 50 μg/mL, respectively.

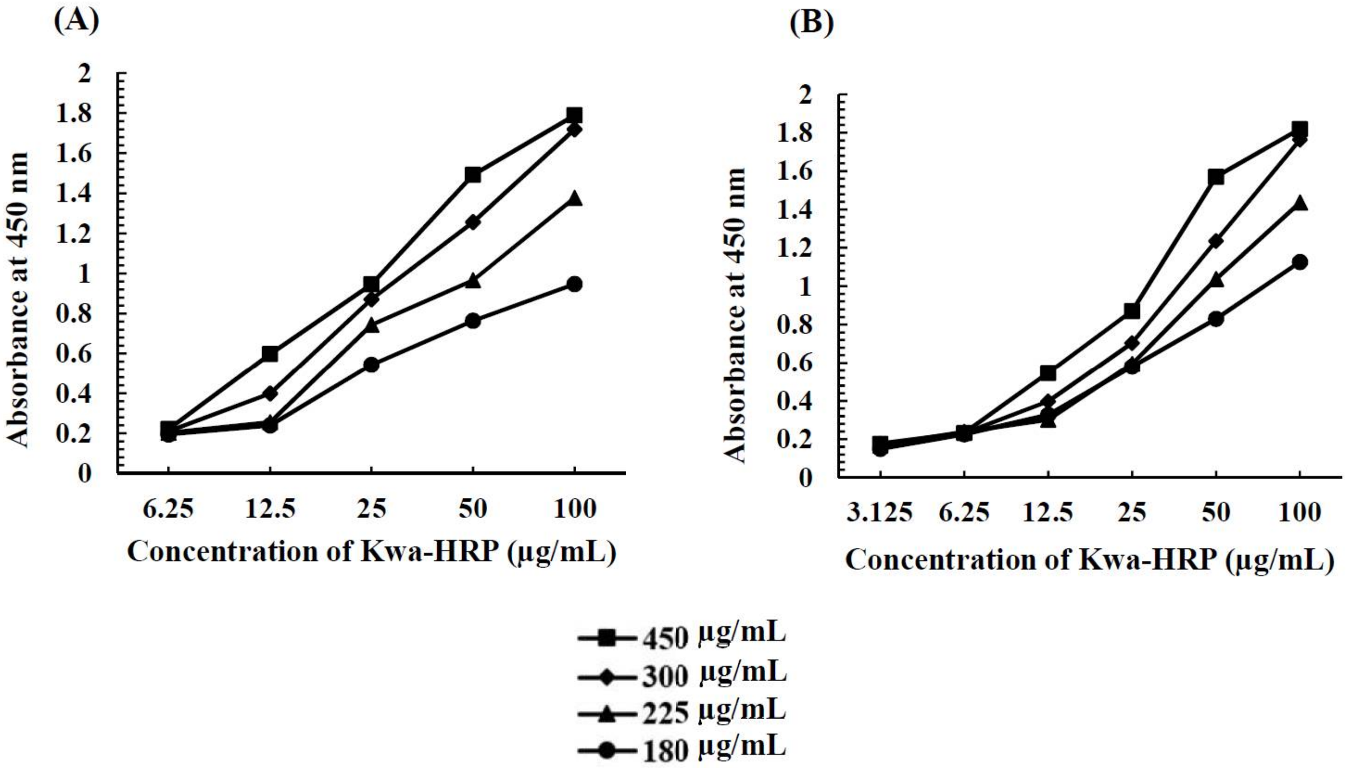

Figure S6. Optimization of concentration of biMAbs ((A) biMAb 2H2 and (B) biMAb 1D2) and Kwa-HRP conjugates. Various biMAb concentrations (180, 225, 300 and 450 μg/mL) and Kwa-HRP conjugates (6.25–100 μg/mL) were applied to ncELBIA.

Table S2. Physicochemical properties of OVA, BSA, HSA, γ-glo, MSA, AVI, TBT, MAb 2H2, and MAb 1D2.

Protein	Accession number	Number of amino acids	Molecular weight	Theoretical pI	Instability index [a]	Aliphatic index [b]	GRAVY [c]
**OVA**	P01012	385	42750	5.19	37	90	–0.006
**BSA**	P02769	583	66433	5.60	40	76	–0.475
**HSA**	P02768	585	66472	5.67	39	76	–0.395
**γ-glo**	P15814	176	19135	9.97	77	60	–0.484
**MSA**	P07724	584	65892	5.53	39	73	–0.432
**AVI**	P02701	128	14343	9.69	22	65	–0.516
**TBT**	P01267	2750	301219	5.50	56	73	–0.257
**MAb 2H2**		1290	142542	8.12	44	65	–0.379
**MAb 1D2**		1292	140796	6.61	36	68	–0.316

[a] A protein with a stability index smaller than 40 is classified as stable, while a protein with a value above 40 is classified as unstable.

[b] Aliphatic index of a protein is defined as the relative volume occupied by aliphatic side chains (Ala, Val, Ile, and Leu). A positive index indicates the increase of the thermostability of globular proteins.

[c] GRAVY index is calculated as the sum of hydropathy values of all of the amino acids divided by the number of residues in the sequence. Protein with negative GRAVY values are hydrophilic, while protein with positive values are hydrophobic.
